# Radiation and Bilateral Spontaneous Pneumothoraces

**DOI:** 10.14740/wjon952e

**Published:** 2015-12-31

**Authors:** Waqas Jehangir, Mohamed Osman, Yazan Vwich, Rafay T. Khan, Shilpi Singh, Abdalla Yousif

**Affiliations:** a Internal Medicine Department, Raritan Bay Medical Center, 530 New Brunswick Ave., Perth Amboy, NJ 08861, USA

**Keywords:** Pneumothorax, Angiosarcoma, Fibrosis, Radiation

## Abstract

Spontaneous pneumothorax can be a rare complication of thoracic radiation therapy with severe consequences. Most of the cases in the medical literature have been described in lymphoma patients receiving radiation therapy. Spontaneous pneumothoraces are divided into two types which consist of primary and secondary. Primary occurs in absence of any known lung condition, while secondary has an underlying lung disease. The etiology of primary spontaneous pneumothorax has not yet been determined but risk factors such as smoking, family history, and male gender have been described unlike that of secondary which is associated with chronic obstructive pulmonary disease (COPD) and other lung conditions. In this report, we discuss the association with radiation therapy and pneumothorax. The pathogenesis of this complication has not fully been elucidated although different mechanisms have been proposed. In this case report, we discuss the findings and management of a female patient treated for angiosarcoma from the scalp with metastasis to the lung that was complicated by pneumothorax.

## Introduction

Primary pneumothorax is an uncommon lung disease that can disrupt respiration in an otherwise healthy individual. It is most commonly caused by rupture of a sub-pleural emphysematous bleb typically located at the apex of the lung. This rupture allows for air from the lung to move into the pleural space and compress the lung. Secondary pneumothorax occurs due to the presence of underlying lung pathology such as chronic obstructive pulmonary disease (COPD) but other causes include but are not limited to an infectious process or malignancy. Much less frequently, spontaneous pneumothorax occurs as a complication of thoracic radiotherapy. In this report, we further illustrate an unusual finding of post-radiation-induced bilateral pneumothorax after the treatment of angiosarcoma.

## Case Report

**Figure 1:**
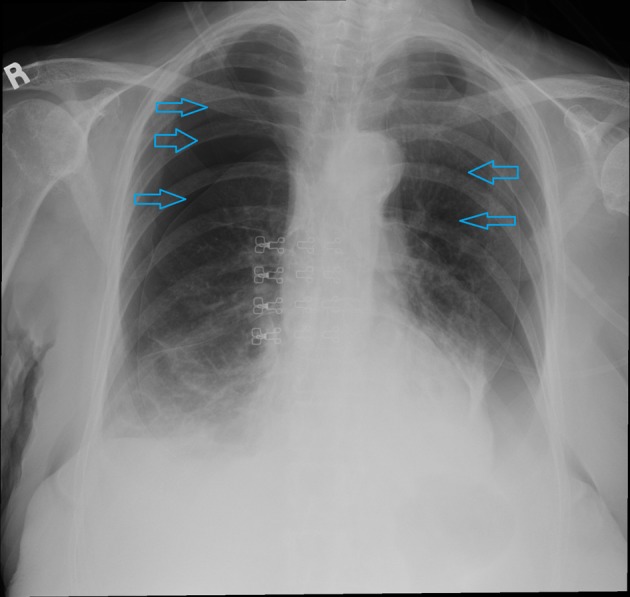
Chest X-ray demonstrating bilateral pneumothoraces.

An 85-year-old Caucasian female nonsmoker with a past medical history significant for angiosarcoma presented to emergency department with a 2-week long history of progressively worsening shortness of breath. The patient’s angiosarcoma originated from the scalp and had extension into the face with metastasis to the lung. The patient previously had surgery to remove the angiosarcoma in the scalp and had reconstruction done. The patient was further treated with radiotherapy for the metastasis to the lung, with the latest treatment administered 2 weeks prior to her presenting to the emergency department. She had received a total of 12 MeV electrons delivered to the site with a dose of 57.2 Gy fractions in 20 fractions over a several week interval. She reported that her shortness of breath had been getting progressively worse and was associated with a productive cough. She denied orthopnea, paroxysmal nocturnal dyspnea, chest pain, palpitations, fevers, chills, or changes in weight. On physical exam, blood pressure was 182/89 mm Hg, pulse was 110 bpm, respiratory rate was 22/min, saturation was 88% on room air, and temperature was 98.3 °F. She was in moderate respiratory distress during examination. There was a large mass on the right cheek and a graft with acanthosis partially healing over the scalp. There were areas of erythema with mild bleeding and raw surfaces on the scalp as well. Lungs examination revealed a resonant chest with a tympanic node overlying the anterior apices, diffuse resonance bilaterally, decreased air entry, and severely diminished breath sounds. Laboratory data showed a hemoglobin of 11 g/dL, hematocrit of 34%, white count of 11,000/μL, platelets of 371,000/μL and an INR of 1. Basic metabolic panel was within normal limits. ECG showed sinus tachycardia. Chest X-ray showed bilateral pneumothoraces at least 50% on the left and 70% on the right (Fig. 1). The patient was diagnosed with radiation-induced spontaneous pneumothorax and bilateral chest tubes were placed by the cardiothoracic surgeon. Patient was discharged home after few days and she was doing fine after 6 months of follow-up.

## Discussion

Pneumothorax has several classifications, some of which consist of tension pneumothorax, non-tension pneumothorax, and spontaneous pneumothorax. Tension pneumothorax is a medical emergency due to rising intrathoracic pressure from progressive air accumulation in the pleural space [1]. On the other hand, non-tension pneumothorax, which is itself divided into open or close (partial) pneumothorax is not as critical as there is no continuous air accumulation and thus no compression of intrathoracic organs [1]. Spontaneous pneumothorax as described earlier is classified depending on the presence of an underlying lung condition.

Spontaneous pneumothorax arises most often in healthy 20 - 40 years old individuals usually due to the rupture of apical blebs on the visceral pleura [2]. In the literature, it has been most frequently reported after the development of radiation fibrosis from mantle irradiation for Hodgkin disease [3]. Pezner et al had demonstrated a frequency of spontaneous pneumothorax in 2.2% of patients with Hodgkin’s disease that were treated with mantle radiation therapy [4]. However, unlike previously documented cases, this case report illustrates a similar effect seen in Hodgkin’s disease treated with radiation in that of a patient with angiosarcoma.

Rupture of subpleural blebs secondary to radiation fibrosis is the proposed mechanism for spontaneous pneumothorax in these patients [5]. The radiation dose to the lung apex has been shown to be higher than that at other points in the chest due to a smaller thoracic diameter superiorly [6]. This results in higher radiation effects and thus fibrosis in normally apical region of the lung which may be the initiating cause.

Pulmonary changes caused by radiotherapy usually begin to occur 8 - 12 weeks following completion of therapy, with slow progression to radiation fibrosis 9 - 12 months following completion of therapy [7]. Sixteen months after radiotherapy has been found to be the average time when spontaneous pneumothorax most often develops [8]. In another study, it was found that patients presented with pneumothorax between 2 and 7 months following completion of therapy [9].

It is important to consider the time frame in order to be prepared if a patient develops symptoms of shortness of breath with a history of radiotherapy in order to not miss this fatal complication. In our patient treatment resulted in full resolution of symptoms. Being prepared and alert when a patient has a history of radiation therapy who is presenting with symptoms of shortness of breath is imperative for clinicians in order to prevent disastrous outcomes. If recurrent symptoms of spontaneous pneumothorax occur, it is possible that inadequately sealing air leaks may be responsible for the persistence and recurrence of pneumothorax in patients previously treated with thoracic radiation [9].

Lastly, it is also imperative to keep in mind that metastasis of malignant tumors to the lungs can also result in spontaneous pneumothorax. Due to the bilateral nature and acute onset in our patient with a known history of radiotherapy, it was unlikely to have been the cause. Nonetheless, any clinical suspicion of metastasis from a primary tumor should warrant a proper and full evaluation.
